# *XDS*

**DOI:** 10.1107/S0907444909047337

**Published:** 2010-01-22

**Authors:** Wolfgang Kabsch

**Affiliations:** a Max-Planck-Institut für Medizinische Forschung, Abteilung Biophysik, Jahnstrasse 29, 69120 Heidelberg, Germany

**Keywords:** *XDS*

## Abstract

The usage and control of recent modifications of the program package *XDS* for the processing of rotation images are described in the context of previous versions. New features include automatic determination of spot size and reflecting range and recognition and assignment of crystal symmetry. Moreover, the limitations of earlier package versions on the number of correction/scaling factors and the representation of pixel contents have been removed. Large program parts have been restructured for parallel processing so that the quality and completeness of collected data can be assessed soon after measurement.

## Abstract 2

The paper describes the software package *XDS* for processing of single crystal diffraction data recorded by the rotation method.

## 1. Functional specification

The program package *XDS* (Kabsch, 1988*a* ▶,*b* ▶, 1993 ▶, 2010 ▶) was developed for the reduction of single-crystal diffraction data recorded on a planar detector by the rotation method using monochromatic X-rays. It includes a set of three programs.

*XDS* accepts a sequence of adjacent non-overlapping rotation images from a variety of imaging-plate, CCD, pixel and multiwire area detectors, infers crystal symmetry and metrics and produces a list of corrected integrated intensities of the reflections occurring in the images in a nearly automatic way. The program assumes that each image covers the same positive amount of crystal rotation and that the rotation axis, incident beam and crystal intersect at one point, but otherwise imposes no limitations on the detector position, on the directions of the rotation axis and incident beam or on the oscillation range covered by each image.

*XSCALE* places the data sets obtained from processing with *XDS* on a common scale, optionally merges them into one or several sets of unique reflections and reports their completeness and the quality of the integrated intensities. It corrects the data for absorption effects, sensitivity variations in the detector plane and radiation damage. Optionally, it can correct reflections individually for radiation damage by extrapolation to their initial intensities at zero dose.

*XDSCONV* converts reflection data files as obtained from *XDS* or *XSCALE* into various formats required by software packages for crystal structure determination. It can generate test reflections or inherit previously selected ones which are used for the calculation of a free *R* factor to monitor the progress of structure refinement.

## 2. *XDS*

*XDS* is organized into eight steps (major subroutines) which are called in succession by the main program. Information is exchanged between the steps by files (see Table 1 ▶), which allows the repetition of selected steps with a different set of input parameters without rerunning the whole program. The files generated by *XDS* are either ASCII-type files that can be inspected and modified using a text editor or binary control images saved as a byte-offset variant of the CBFlib format (Bernstein & Hammersley, 2005 ▶; Bernstein & Ellis, 2005 ▶). Such images are indicated by the file-name extension .cbf and can be looked at using the open-source program *XDS-Viewer* written by Michael Hoffer.

All files have a fixed name defined by *XDS*, which makes it mandatory to process each data set in a newly created directory in order to avoid name clashes. Clearly, one should not run more than one *XDS* job at a time in the same directory. Output files affected by rerunning selected steps (see Table 1 ▶) should also first be given another name if their original contents are meant to be saved.

Data processing begins by copying an appropriate input file into the new directory. Input-file templates are provided with the *XDS* package for a number of frequently used data-collection facilities. The copied input file must be renamed XDS.INP and edited to provide the correct parameter values for the actual data-collection experiment. All parameters in XDS.INP are named by keywords containing an equals sign as the last character and many of them will be mentioned here in context in order to clarify their meaning. Execution of *XDS* (JOB=XDS) invokes each of the eight program steps as described below. The results and diagnostics from each step are saved in files with the extension .LP attached to the program-step name. These files should always be studied carefully to see whether processing was satisfactory or, in the case of failure, to find out what could have gone wrong.

### 2.1. *XYCORR*

This program step calculates a lookup table of additive spatial corrections at each detector pixel which is stored in the files X-CORRECTIONS.cbf, Y-CORRECTIONS.cbf. Often, the data images have already been corrected for geometrical distortions, in which case *XYCORR* produces tables of zeros.

For spiral read-out imaging-plate detectors the small corrections resulting from radial (ROFF=) and tangential (TOFF=) offset errors of the scanner are computed.

For some multiwire and CCD detectors that deliver geo­metrically distorted images, corrections are derived from a calibration image (BRASS_PLATE_IMAGE=file name). This image displays the response to a brass plate containing a regular grid of holes which is mounted in front of the detector and illuminated by an X-ray point source. Clearly, the source must be placed exactly at the location to be occupied by the crystal during the actual data collection, as photons emanating from the calibration source are meant to simulate all possible diffracted beam directions. For visual control, spots that have been located and accepted from the brass-plate image by *XYCORR* are marked in the file FRAME.cbf.

The following problems can be encountered in this step.

(i) A misplaced calibration source can lead to an incorrect lookup table, impairing the correct prediction of the observed diffraction pattern in subsequent program steps.
(ii) An underexposed calibration image can result in an incomplete and unreliable list of calibration spots.

### 2.2. *INIT*

*INIT* determines three lookup tables, saved as the files BLANK.cbf, GAIN.cbf and BKGINIT.cbf, that are required by the subsequent processing steps for classifying pixels in the data images as background or belonging to a diffraction spot (‘strong’ pixels). These tables should be inspected with the *XDS-Viewer* program.

BLANK.cbf contains a lookup table of the detector noise. It is determined from a specific image recorded in the absence of X-rays (DARK_CURRENT_IMAGE=) or is assumed to be a constant derived from the mean recorded value in each corner of the data images.

GAIN.cbf codes for the expected variation of the pixel contents in the background region of a data image. The variance of the contents of a pixel in the background region is GAIN·(pixel contents − detector noise). The variance is determined from the scatter of pixel values within a rectangular box (NBX=, NBY=) of size (2·NBX + 1)·(2·NBY + 1) centred at each image pixel in succession. The table GAIN.cbf is used to distinguish background pixels from ‘strong’ pixels that are part of a diffraction spot.

BKGINIT.cbf estimates the initial background at each pixel from a few data images specified by the input parameter BACKGROUND_RANGE=. The lookup table is obtained by adding the X-ray background from each image. Shaded regions on the detector (*i.e.* from the beamstop), pixels outside a user-defined circular region (TRUSTED_REGION=) or pixels with an undefined spatial correction value are classified as untrustworthy and marked by −3.

The following problem can be encountered in this step. Some detectors with insufficient protection from electromagnetic pulses may generate badly spoiled images whose inclusion leads to a completely wrong X-ray background table. These images can be identified in INIT.LP by their un­expected high mean pixel contents and this step should be repeated with a different set of images.

### 2.3. *COLSPOT*

*COLSPOT* locates strong diffraction spots occurring in a subset of the data images and saves their centroids in the file SPOT.XDS. The data subset is defined by contiguous image number ranges, where each range is specified by the keyword SPOT_RANGE=. As described in Kabsch (2010 ▶), spots are defined as sets of ‘strong’ pixels that are adjacent in three dimensions. The classification of ‘strong’ pixels is controlled by the decision constants STRONG_PIXEL= and BACKGROUND_PIXEL=. If the total number of ‘strong’ pixels occurring in the specified data images exceeds the upper limit as given by the input parameter MAXIMUM_NUMBER_OF_STRONG_PIXELS=, the weaker ones are discarded. A spot is accepted if it contains a minimum number of ‘strong’ pixels (MINIMUM_NUMBER_OF_PIXELS_IN_A_SPOT=) and if the spot centroid is sufficiently close to the location of the strongest pixel in the spot (SPOT_MAXIMUM-CENTROID=).

The following problem can be encountered in this step. Sharp edges such as ice rings in the images can lead to an excessive number of ‘strong’ pixels being erroneously classified as contributing to diffraction spots. These aliens could prevent *IDXREF* from recognizing the crystal lattice.

### 2.4. *IDXREF*

*IDXREF* uses the initial parameters describing the diffraction experiment as provided by XDS.INP and the observed centroids of the spots from the file SPOT.XDS to find the orientation, metric and symmetry of the crystal lattice and refines all or a specified subset of these parameters [input parameter REFINE(IDXREF)=] . On return, the complete set of parameters are saved in the file XPARM.XDS and the original file SPOT.XDS is replaced by a file of identical name, now with indices attached to each observed spot. Spots not belonging to the crystal lattice are given indices 0, 0, 0. *XDS* considers the run to be successful if the coordinates of at least 70% of the given spots can be explained with reasonable accuracy (input parameter MAXIMUM_ERROR_OF_SPOT_POSITION=); otherwise, *XDS* will stop with an error message. Alien spots often arise because of the presence of ice or small satellite crystals and continuation of data processing may still be meaningful. In this case, *XDS* is called again with an explicit list of the subsequent steps specified in XDS.INP (input parameter JOB=DEFPIX XPLAN INTEGRATE CORRECT).

*IDXREF* uses the methods described in Kabsch (1993 ▶, 2010 ▶) to determine a crystal lattice that explains the observed locations of the diffraction spots listed in the file SPOT.XDS. Firstly, a reciprocal-lattice vector referring to the unrotated crystal is computed from each observed spot centroid. Differences between any two reciprocal-lattice vectors that are above a specified minimal length (SEPMIN=) are accumulated in a three-dimensional histogram. These difference vectors will form clusters in the histogram, since there are many different pairs of reciprocal-lattice vectors of nearly identical vector difference. The clusters are found as maxima in the smoothed histogram (CLUSTER_RADIUS=) and a basis of three linearly independent cluster vectors is selected that allows all other cluster vectors to be expressed as nearly integral multiples of small magnitude with respect to this basis. The basis vectors and the 60 most populated clusters with attached indices are listed in IDXREF.LP. If many of the indices deviate significantly from integral values, the program is unable to find a reasonable lattice basis and all further processing will be meaningless.

If the space group and unit-cell parameters are specified, a reduced cell is derived and the reciprocal-basis vectors found above are reinterpreted accordingly; otherwise, a reduced cell is determined directly from the reciprocal basis. The parameters of the reduced cell, the coordinates of the reciprocal-basis vectors and their indices with respect to the reduced cell are reported.

Based on the orientation and metric of the reduced cell now available, *IDXREF* indexes up to 3000 of the strongest spots using the local-indexing method. This method considers each spot as a node of a tree and identifies the largest subtree of nodes which can be assigned reliable indices. The number of reflections in the ten largest subtrees is reported and usually shows a dominant first tree corresponding to a single lattice, whereas alien spots are found in small subtrees. Reflections in the largest subtree are used for initial refinement of the basis vectors of the reduced cell, the incident-beam wavevector and the origin of the detector, which is the point in the detector plane nearest to the crystal. Experience has shown that the detector origin and the direction of the incident beam are often specified with insufficient accuracy, which could easily lead to a misindexing of the reflections by a constant offset. For this reason, *IDXREF* considers alternative choices for the index origin and reports their likelihood of being correct. The parameters controlling the local indexing are INDEX_ERROR=, INDEX_MAGNITUDE=, INDEX_QUALITY= (corresponding to ∊, ϕ and 1 − ℓ_min_ in Kabsch, 2010 ▶) and INDEX_ORIGIN=*h*_0_, *k*_0_, *l*_0_, which is added to the indices of all reflections in the tree. After initial refinement based on the reflections in the largest subtree, all spots which can now be indexed are included. Usually, the detector distance and the direction of the rotation axis are not refined, but if the spots were extracted from images covering a large range of total crystal rotation then better results are obtained by including these parameters in the refinement [REFINE(IDXREF)=] .

The refined metric parameters of the reduced cell are used to test each of the 44 possible lattice types as described in Kabsch (2010 ▶). For each lattice type, *IDXREF* reports the likelihood of its being correct and the conventional unit-cell parameters. The program step concludes with an overview of possible lattice symmetries, but makes no automatic decision for the space group. If the crystal symmetry is unknown, *XDS* will continue data processing with the crystal being described by its reduced-cell basis vectors and triclinic symmetry. Space-group assignment is postponed to the last program step, *CORRECT*, when integrated intensities are available.

The following problems can be encountered in this step.

(i) The indices of many difference-vector clusters deviate significantly from integral values. This can be caused by incorrect input parameters, such as rotation axis, oscillation angle or detector position, by a large fraction of alien spots in SPOT.XDS, by placing the detector too close to the crystal or by an inappropriate choice of the parameters SEPMIN= and CLUSTER_RADIUS= in densely populated images.
(ii) Indexing and refinement is unsatisfactory despite well indexed difference-vector clusters. This is probably caused by the selection of an incorrect index origin and *IDXREF* should be rerun with plausible alternatives for INDEX_ORIGIN= after a visual check of a data image with *XDS-Viewer*.
(iii) Despite successful indexing and refinement, *IDXREF* stops with the error message INSUFFICIENT PERCENTAGE OF INDEXED REFLECTIONS, complaining that less than 70% of the given spots could be explained. Alien spots often arise because of the presence of ice or small satellite crystals and continuation of data processing may still be meaningful. To continue data processing, just specify the missing processing steps in XDS.INP by JOB=DEFPIX XPLAN INTEGRATE CORRECT and call *XDS* again.

### 2.5. *DEFPIX*

*DEFPIX* recognizes regions in the initial background table (file BKGINIT.cbf) that are obscured by intruding hardware and marks the shaded pixels as untrusted. In addition, pixels that are outside a user-defined resolution range (INCLUDE_RESOLUTION_RANGE=) are marked and eliminated from the trusted region. The marked background table that is thus obtained is saved in the file BKGPIX.cbf which is needed by the subsequent program steps. To recognize the obscured regions in the initial background, *DEFPIX* generates a control image (file ABS.cbf) that contains values of around 10 000 for unshaded pixels and lower values for shaded pixels. The classification of the pixels into reliable and untrusted pixels is based on the two input parameters VALUE_RANGE_FOR_TRUSTED_DETECTOR_PIXELS= (default 6000 30 000) and INCLUDE_RESOLUTION_RANGE= (default 20.0 0.0). Pixels in the table ABS.cbf with a value outside the ranges specified by the two parameters are marked unreliable (by −3) in the background table BKGPIX.cbf.

The following problem can be encountered in this step. If the parameter VALUE_RANGE_FOR_TRUSTED_DETECTOR_PIXELS= specifies a value range that is too narrow, ‘good’ regions will erroneously be excluded from the trusted detector region. Check BKGPIX.cbf with the *XDS-Viewer* program and if necessary repeat the *DEFPIX* step with more appropriate values.

### 2.6. *XPLAN*

*XPLAN* supports the planning of data collection. It is based upon information provided by the input files XPARM.XDS and BKGPIX.cbf, both of which become available on processing a few test images with *XDS*. *XPLAN* estimates the completeness of new reflection data expected to be collected for each given starting angle and total crystal rotation and reports the results for a number of selected resolution shells in the file XPLAN.LP. To minimize the recollection of data, the name of a file containing already measured reflections can be provided by the input parameter REFERENCE_DATA_SET=. 

The following problems can be encountered in this step.

(i) Incorrect results may occur for some space groups, *i.e. P*4_2_, if the unit cell determined by *XDS* from processing a few test images implicates reflection indices that are inconsistent with those from the reference data set. However, the correct cell choice can be found by using the old data as a reference and repeating *CORRECT* with the appropriate reindexing transformation, followed by copying GXPARM.XDS to XPARM.XDS. The same applies if *IDXREF* was run for an unknown space group and then reindexed in *CORRECT*.
(ii) *XPLAN* ignores potential reflection overlap owing to the finite oscillation range covered by each image.

### 2.7. *INTEGRATE*

*INTEGRATE* determines the intensity of each reflection predicted to occur in the rotation data images (DATA_RANGE=) and saves the results in the file INTEGRATE.HKL.

The diffraction parameters needed to predict the reflection positions are initially provided by the file XPARM.XDS. These parameters are either kept constant or refined periodically using strong diffraction spots encountered in the data images. Whether refinement should be carried out at all and which parameters are to be refined can be specified by the user [input parameter REFINE(INTEGRATE)=]. The centroids of the strong spots in the data images are computed from pixels that exceed the background by a given multiple of standard deviations (input parameters SIGNAL_PIXEL=, BACKGROUND_PIXEL=). Strong spots are used in the refinement if their centroids are reasonably close to their calculated position (input parameter MAXIMUM_ERROR_OF_SPOT_POSITION=).

For determination of the intensity, approximate values describing the extension and the form of the diffraction spot must be specified. The shapes of all spots become very similar when the contents of each of their contributing image pixels is mapped onto a three-dimensional coordinate system, specific for each reflection, which has its origin on the surface of the Ewald sphere at the terminus of the diffracted beam wavevector (see Kabsch, 2010 ▶). The transformed spot can roughly be described as a Gaussian involving two parameters: the standard deviations of the reflecting range σ_M_ (input parameter REFLECTING_RANGE_E.S.D.=σ_M_) and the beam divergence σ_D_ (input parameter BEAM_DIVERGENCE_E.S.D.=σ_D_). This leads to an integration region around the spot that is defined by the parameters δ_M_ (REFLECTING_RANGE=) and δ_D_ (BEAM_DIVERGENCE=), which are typically chosen to be 6–10 times larger than σ_M_ and σ_D_, respectively. Appropriate values for these parameters are determined automatically by *XDS* (Kabsch, 2010 ▶); the user has the option to override the automatic assignments.

Integration is carried out by a two-step procedure. In the first pass, spot templates are generated by superimposing the profiles of strong reflections after their mapping to the Ewald sphere. Grid points with a value above a minimum percentage of the maximum in the template (parameter CUT=) are marked for inclusion in the final integration. To allow for variations in their shape, profile templates are generated from reflections located at nine regions of equal size covering the detector surface and additional sets of nine to cover equally sized (parameter DELPHI=) batches of images. The actual integration is carried out in the second pass by profile fitting with respect to the spot shape determined in the first pass. Incomplete reflections below a minimum percentage of the observed reflection intensity (parameter MINPK=) will be discarded. Otherwise, the missing intensity is estimated from the learned reflection profiles.

On return from the* INTEGRATE* step, all spots expected to occur in the last data image are encircled and the modified image is saved as the file FRAME.cbf for inspection.

The following problems can be encountered in this step.

(i) Off-centred profiles indicate incorrectly predicted reflection positions by using the parameters provided by the file XPARM.XDS (*i.e.* misindexing by using a wrong origin of the indices), crystal slippage or change in the incident-beam direction.
(ii) Profiles extending to the borders of the box indicate too-small values of the parameters BEAM_DIVERGENCE= or REFLECTING_RANGE=. This leads to incorrect integrated intensities because of truncated reflection profiles and un­reliable background determination.
(iii) Display of the file FRAME.cbf shows spots which are not encircled. If these unexpected reflections are not close to the spindle and are not ice reflections, then it is likely that the parameters provided by the file XPARM.XDS are wrong.

### 2.8. *CORRECT*

*CORRECT* applies correction factors to the intensities and standard deviations of all reflections found in the file INTEGRATE.HKL, determines the space group if unknown and refines the unit-cell parameters, reports the quality and com­pleteness of the data set and saves the final integrated intensities in the file XDS_ASCII.HKL. Some of the employed algorithms are new and are described in Kabsch (2010 ▶).

*CORRECT* accepts reflections from the file INTEGRATE.HKL that are

(i) recorded (parameter MINPK=) on specified images (parameter DATA_RANGE=);
(ii) within a given resolution range (parameter INCLUDE_RESOLUTION_RANGE=);
(iii) outside ice rings (parameter EXCLUDE_RESOLUTION_RANGE=);
(iv) not overloaded (parameter OVERLOAD=); and
(v) not marked for exclusion in the file REMOVE.HKL.

Thus, the user has the option to exclude unreliable reflections from the final data set by repeating the *CORRECT* step with appropriate parameter values.

The intensities of the accepted reflections are first corrected for effects arising from polarization of the incident beam (parameters FRACTION_OF_POLARIZATION=, POLARIZATION_PLANE_NORMAL=) and absorption effects (parameters AIR=, SILICON=, SENSOR_THICKNESS=) arising from differences in path lengths of the diffracted beam. These corrections do not depend on knowledge of the space group.

The integrated intensities of the reflections in the file INTEGRATE.HKL may or may not have been indexed in the correct space group; for the purpose of integration, it is important only that all reflections occurring in the data images have been indexed with respect to some unit-cell basis and that their locations on the images were hit exactly. The correct reflection indices in the true space group are always a linear transformation of the original indices used in INTEGRATE.HKL. All lattices consistent with the locations of the reflections saved in INTEGRATE.HKL (decision parameters MAX_CELL_AXIS_ERROR=, MAX_CELL_ANGLE_ERROR=) and their corresponding linear transformations are printed to provide a useful overview similar to that shown in IDXREF.LP.

If the space group is not specified, *XDS* proposes one of the enantiomorphous space groups without screw axes that is compatible with the observed lattice symmetry and explains the intensities of a subset of the reflections (parameter TEST_RESOLUTION_RANGE=) at an acceptable *R*_meas_ (Diederichs & Karplus, 1997 ▶; Weiss, 2001 ▶) using a minimum number of unique reflections. The criteria for an acceptable *R*_meas_ are controlled by the decision parameters MIN_RFL_Rmeas= and MAX_FAC_Rmeas=. 

The user can always override the automatic decisions by specifying the correct space-group number (parameter SPACE_GROUP_NUMBER=) and unit-cell parameters (parameter UNIT_CELL_CONSTANTS=) in XDS.INP and repeating the *CORRECT* step. This provides a simple way to rename orthorhombic unit-cell parameters, which often becomes necessary if screw axes are present. In addition, the user has the option to specify the following in XDS.INP:

(i) a reference data set (parameter REFERENCE_DATA_SET=),
(ii) a reindexing transformation (parameter REIDX=) and
(iii) three basis vectors if known from processing a previous data set taken at the same crystal orientation in a multi-wavelength experiment (parameters UNIT_CELL_A-AXIS=, UNIT_CELL_B-AXIS=, UNIT_CELL_C-AXIS=).

The possibility of comparing the new data with a reference data set is particularly useful for resolving the issue of alternative settings of polar or rhombohedral cells (such as *P*4, *P*6 and *R*3). Also, reference data are quite useful for recognizing misindexing or for testing potential heavy-atom derivatives.

For refinement of the unit-cell parameters [parameter REFINE(CORRECT)=], *CORRECT* uses a subset of the accepted reflections whose observed centroid is sufficiently close to the predicted spot position (parameter MAXIMUM_ERROR_OF_SPOT_POSITION=). The refined set of parameters is saved in the file GXPARM.XDS, which has an identical layout to the file XPARM.XDS produced by *IDXREF*. If the crystal has not slipped during data collection, these parameters are quite accurate.

Other correction factors (parameter CORRECTIONS=) which partially compensate for radiation damage, absorption effects and variations in the sensitivity of the detector surface are determined from the symmetry-equivalent reflections usually found in the data images. The corrections are chosen such that the integrated intensities of symmetry-equivalent reflections come out as similar as possible. The user may control application of the various corrections by specifying the parameter CORRECTIONS= by a combination of the key­words DECAY MODULATION ABSORPTION. Whether Friedel pairs are considered as symmetry-equivalent reflections in the calculation of the correction factors depends on the values of the two parameters STRICT_ABSORPTION_CORRECTION= and FRIEDEL’S_LAW=. The number of correction factors is controlled by the input parameters MINIMUM_I/SIGMA=, NBATCH= and REFLECTIONS/CORRECTION_FACTOR=. 

The residual scatter in intensity of symmetry-equivalent reflections is used to estimate their standard deviations. Here, the initial estimate *v*_0_(*I*) (obtained from the *INTEGRATE* step) for the variance of the reflection intensity *I* is replaced by *v*(*I*) = *a*[*v*_0_(*I*) + *bI* ^2^]. The two constants *a* and *b* are chosen to minimize discrepancies between *v*(*I*) and the variance estimated from sample statistics of symmetry-related reflections. Based on the more realistic error estimates for the intensities, outliers are recognized by comparison with other symmetry-equivalent reflections. These outliers are included in the main output file XDS_ASCII.HKL, in which they are marked by a negative sign attached to the estimated standard deviations of their intensity. Classification of a reflection as a misfit is con­trolled by a decision constant which has the default value of WFAC1=1.5. Specification of a lower value such as  WFAC1=1.0 by the user will lead to an increasing number of misfits and lower *R* factors as outliers are not included in the reported statistics.

Data quality as a function of resolution is described by the agreement of intensities of symmetry-related reflections and quantified by the *R* factors *R*_merge_ and the more robust indicator *R*_meas_ (Diederichs & Karplus, 1997 ▶; Weiss, 2001 ▶). These *R* factors as well as the intensities of all reflections with indices of type *h*00, 0*k*0 and 00*l* and those expected to be systematically absent provide important information for identification of the correct space group. Clearly, large *R* factors or many rejected reflections or large observed intensities for reflections that are expected to be systematically absent suggest that the assumed space group or indexing is incorrect. The presence or absence of anomalous scatterers is specified by the parameter FRIEDEL’S_LAW=. Finally, *CORRECT* analyzes the distribution of reflection intensities as a function of their resolution and reports outliers from the Wilson plot. Often, these aliens arise from ice rings in the data images. To suppress the un­wanted reflections from the final output file XDS_ASCII.HKL, the user copies them to a file named REMOVE.HKL in the current directory and repeats the *CORRECT* step.

The following problems can be encountered in this step.

(i) Incomplete data sets may lead to wrong conclusions about the space group, as some of its symmetry operators might not be involved in the *R*-factor calculations.
(ii) Often, the *CORRECT* step is repeated several times. It should be remembered that *XDS* overwrites earlier versions of the output files XDS_ASCII.HKL, GXPARM.XDS *etc*.

## 3. *XSCALE*

The scaling program *XSCALE*

(i) puts one or more files obtained from data processing with *XDS* on a common scale and reports the completeness and quality of the data sets;
(ii) offers a choice of either combining symmetry-equivalent observations into a single unique reflection or saving the scaled but unmerged observations in the output file;
(iii) allows several output files that are placed on the same scale, a feature that is recommended for MAD data sets taken from the same crystal at different wavelengths;
(iv) determines correction factors that partially compensate for absorption effects, sensitivity variations in the detector plane and radiation damage; and
(v) can correct reflections individually for radiation damage (Diederichs *et al.*, 2003 ▶).

The program uses a new fast algorithm (Kabsch, 2010 ▶) and imposes no limitations on the number of data sets or scaling/correction factors. The easiest way to run *XSCALE* is to copy a template input file named XSCALE.INP to a new directory and to replace the parameter values by the appropriate values describing the actual scaling run. The input parameters may be given in arbitrary order, except for the parameters defining the input and output reflection files (INPUT_FILE=, OUTPUT_FILE=). Here, an output file is defined first by the parameter OUTPUT_FILE= that will include the scaled and merged reflections from all following input files specified by the parameters INPUT_FILE= until the next occurrence of OUTPUT_FILE= in XSCALE.INP. An arbitrary number of output files can be specified (together with their set of input files) in a single run of *XSCALE*. All output files are then on the same scale, which is a useful program feature for MAD data sets.

The reflections in each output file will be unmerged and Friedel pairs will be considered to be different if this holds for all of the input data sets unless explicitly redefined by the parameters MERGE= and FRIEDEL’S_LAW=. Moreover, each output file accepts an additional parameter that controls how the Friedel pairs of the input files are treated in the calculation of the absorption correction factors. If STRICT_ABSORPTION_CORRECTION=FALSE, Friedel pairs are treated as symmetry-equivalent reflections in these calculations, which could lead to an underestimate of the anomalous differences in the presence of anomalous scatterers. Friedel pairs are only treated as different reflections in the calculations if STRICT_ABSORPTION_CORRECTION=TRUE and FRIEDEL’S_LAW=FALSE.

For each input file, a resolution window for accepting reflections (INCLUDE_RESOLUTION_RANGE=), the extent of absorption corrections (CORRECTIONS=DECAY MODULATION ABSORPTION) and the number of correction factors (NBATCH=) can be specified. Finally, each input data set can be corrected for radiation damage by specifying the name of the crystal the data set was obtained from (CRYSTAL_NAME=). Specification of this parameter implicates zero-dose extrapolation of individual reflection intensities to compensate for the effects of radiation damage experienced by the crystal so far (see Diederichs *et al.*, 2003 ▶).

Each resulting scaled data set is of XDS_ASCII format. It can be converted into a CCP4-style multi-record MTZ file using the copy feature of the program *POINTLESS* (Evans, 2006 ▶) available from the web (ftp://ftp.ccp4.ac.uk/ccp4/6.0.2/prerelease/pointless.html) or converted by *XDSCONV* into the format required by various structure-solution packages.

## 4. *XDSCONV*

*XDSCONV* accepts reflection-intensity data files as produced by *XSCALE* or *CORRECT* and converts them into the format required by software packages for structure determination. *XDSCONV* estimates structure-factor moduli based on the assumption that the intensity data set obeys Wilson’s distribution and uses a Bayesian approach to statistical inference as described by French & Wilson (1978 ▶). The output file generated may inherit the test reflections previously used to calculate a free *R* factor (Brünger, 1992 ▶) or may contain new test reflections selected by *XDSCONV*.

## 5. Parallelization of *XDS*

In order to efficiently use modern multiprocessor hardware, a major effort has been undertaken to replace the original code of *XDS* by routines that can run concurrently with very little need for synchronization. As described above, data processing by *XDS* is organized into eight steps that must be executed in a fixed order since the result of each step is needed as input for the subsequent ones. Thus, the only way to speed up processing is to make each step faster.

The most computationally intensive steps are *COLSPOT* and *INTEGRATE* and, to a lesser degree, the routine that refines diffraction parameters in *IDXREF* and *CORRECT*. Thus, the highest savings in wall clock time are expected to result from changing these routines so that each one can make efficient use of the multiprocessor hardware. Two methods can be used (simultaneously) to speed up data processing.

In the first method, *XDS* divides the set of data images into approximately equal portions, calls a shell script that starts an independent job for processing each portion of images by the computer cluster and waits until all jobs have finished. The number of such independent jobs can be limited by the user (MAXIMUM_NUMBER_OF_JOBS=); up to 99 jobs are allowed. This method works even if the processors do not share the same address space since the jobs are independent processes that do not communicate at all.

The second method uses OpenMP to control execution by a team of threads and relies on a shared-memory multiprocessor platform. This allows the program to exploit data parallelism at a more fine-grained level to speed up refinements and routines for setting up and solving systems of linear equations. The maximum number of threads that can be employed by the parallel version of *XDS* (xds_par, xscale_par) can be limited by the user (MAXIMUM_NUMBER_OF_PROCESSORS=); up to 32 processors can be used. OpenMP has been chosen for execution control because it hardly adds to the complexity of the program code and most importantly does not require the maintenance of separate versions of the source code depending on whether the program is intended for execution by a team of processors or just by a single CPU. Moreover, OpenMP has become the *de facto* standard and compilers accepting OpenMP directives are available for most shared-memory multiprocessor platforms.

The new version of *COLSPOT* comprises an initial part, a concurrent procedure and a final part. After initialization each available processor is kept busy analyzing its share of rotation images for strong pixels, which are saved in a processor-specific file. In the final sequential part of *COLSPOT* all files resulting from the concurrent computations are read and the location addresses, image running numbers and signal values of the strong pixels are stored in a hash table. Strong pixels belonging to the same spot can be located rapidly in this table and the centroids of the spots are saved in the final output file from this step.

For the *INTEGRATE* step, the rotation images are divided into approximately equal portions for independent processing under control by a shell script according to the first method described above. When all jobs have finished, the integrated intensities from each independent file are joined. Minor problems could occur for reflections that receive intensity contributions from images that have been processed by different jobs. Compared with processing as a single job, the observed intensity differences are small and disappear if the different jobs use identical reference profiles and diffraction parameters to predict spot locations [to avoid refinements, specify REFINE(INTEGRATE)=!].

In addition, each of the independent jobs can be executed by a team of processors controlled by OpenMP. The rotation images analyzed by each job are split into a sequence of batches of consecutive images that cover a total rotation range that is large enough to accommodate the integration domain. The batches are evaluated in strictly sequental order; parallel processing is confined to images within each batch.

The restructured routine for the *INTEGRATE* step consists of code regions for parallel execution interspersed by sequential sections. After initialization, strong reflections and their mean size and extent are determined concurrently. The diffraction parameters are refined in parallel processing mode based on the observed spot locations. In the following sequential section a database is generated containing information about all reflections occurring in this batch of images. A subset of strong reflections is also identified that is useful for the subsequent reflection-profile learning pass. The mean profile of these reflections is determined concurrently in a second pass through the images in the batch. Reflection integration by profile fitting is carried out in parallel in the third cycle through the batch. In the final sequential step the results from each job, which have been saved in files, are harvested and intensity contributions to the same reflection from adjacent batches are merged.

## 6. Availability

Documentation and executable versions of the *XDS* package for widely used computer systems running under Linux or OSX can be obtained from the *XDS* homepage (http://xds.mpimf-heidelberg.mpg.de/) free of charge for use by academics for noncommercial applications. Additional information can be found at http://strucbio.biologie.uni-konstanz.de/xdswiki/index.php/XDS.

For looking at rotation data images and control images generated by *XDS*, an open-source program *XDS-Viewer* written by Michael Hoffer can be obtained from http://xds-viewer.sourceforge.net under the GNU General Public License.

A graphical interface *XDSi* (Kursula, 2004 ▶) is available (http://cc.oulu.fi/~pkursula/xdsi.html) that simplifies the operation of *XDS*.

## Figures and Tables

**Table 1:** Information exchange between program steps of *XDS*

Program step	Input files	Output files
*XYCORR*	XDS.INP	XYCORR.LP
		X-CORRECTIONS.cbf
		Y-CORRECTIONS.cbf
		FRAME.cbf
		
*INIT*	XDS.INP	INIT.LP
	X-CORRECTIONS.cbf	BKGINIT.cbf
	Y-CORRECTIONS.cbf	BLANK.cbf
		GAIN.cbf
		
*COLSPOT*	XDS.INP	COLSPOT.LP
	X-CORRECTIONS.cbf	SPOT.XDS
	Y-CORRECTIONS.cbf	
	BLANK.cbf	
	BKGINIT.cbf	
	GAIN.cbf	
		
*IDXREF*	XDS.INP	IDXREF.LP
	SPOT.XDS	SPOT.XDS
		XPARM.XDS
		
*DEFPIX*	XDS.INP	DEFPIX.LP
	X-CORRECTIONS.cbf	BKGPIX.cbf
	Y-CORRECTIONS.cbf	ABS.cbf
	BKGINIT.cbf	
	XPARM.XDS	
		
*XPLAN*	XDS.INP	XPLAN.LP
	XPARM.XDS	
	BKGPIX.cbf	
	X-CORRECTIONS.cbf	
	Y-CORRECTIONS.cbf	
		
*INTEGRATE*	XDS.INP	INTEGRATE.LP
	X-CORRECTIONS.cbf	INTEGRATE.HKL
	Y-CORRECTIONS.cbf	FRAME.cbf
	BLANK.cbf	
	BKGPIX.cbf	
	GAIN.cbf	
	XPARM.XDS	
		
*CORRECT*	XDS.INP	CORRECT.LP
	INTEGRATE.HKL	XDS_ASCII.HKL
	REMOVE.HKL	GXPARM.XDS
	X-CORRECTIONS.cbf	DX-CORRECTIONS.cbf
	Y-CORRECTIONS.cbf	DY-CORRECTIONS.cbf
		*GX*-CORRECTIONS.cbf
		GY-CORRECTIONS.cbf
		ABSORP.cbf
		DECAY.cbf
		MODPIX.cbf
